# Cellular expression profiles of Epstein-Barr virus-transformed B-lymphoblastoid cell lines

**DOI:** 10.3892/br.2020.1379

**Published:** 2020-10-22

**Authors:** Arkom Chaiwongkot, Nakarin Kitkumthorn, Ratakorn Srisuttee, Supranee Buranapraditkun

Biomed Rep 13:Article number 43, 2020; DOI: 10.3892/br.2020.1350

After the publication of the above paper, the authors noted that the affiliation for the third named author (Ratakorn Srisuttee) was presented incorrectly: Specifically, the affiliation should have been written as Faculty of Medicine, King Mongkut's Institute of Technology Ladkrabang, Bangkok 10520 (i.e., “Ladkrabang” should have been included with the affiliation). Therefore, the affiliations for this paper should have appeared as follows:

ARKOM CHAIWONGKOT^1,2^, NAKARIN KITKUMTHORN^3^, RATAKORN SRISUTTEE^4^ AND SUPRANEE BURANAPRADITKUN^5,6^

^1^Applied Medical Virology Research Unit; ^2^Department of Microbiology, Faculty of Medicine, Chulalongkorn University, Bangkok 10330; ^3^Department of Oral Biology, Faculty of Dentistry, Mahidol University, Bangkok 10400; ^4^Faculty of Medicine, King Mongkut's Institute of Technology Ladkrabang, Bangkok 10520; ^5^Division of Allergy and Clinical Immunology, Department of Medicine; ^6^Center of Excellence in Vaccine Research and Development (Chula Vaccine Research Center-Chula VRC), Faculty of Medicine, Chulalongkorn University, Bangkok 10330, Thailand

The authors regret that this error with the author affiliations for Ratakorn Srisuttee was not noticed prior to the publication of their paper, and apologize for any inconvenience caused.

